# Building a national eye-care service in post-conflict Timor-Leste

**DOI:** 10.2471/BLT.18.212506

**Published:** 2018-08-27

**Authors:** Kristof Wing, Gwyn Low, Manoj Sharma, Frenky De Jesus, Belmerio Jeronimo, Nitin Verma

**Affiliations:** a College of Health and Medicine, University of Tasmania, 17 Liverpool St, Hobart, Tasmania 7000, Australia.; b Royal Australasian College of Surgeons, Melbourne, Australia.; c Department of Ophthalmology, Hospital Nacional Guido Valadares, Dili, Timor-Leste.

## Abstract

**Problem:**

Violent conflict left Timor-Leste with a dismantled health-care workforce and infrastructure after 2001. The absence of existing health and tertiary education sectors compounded the challenges of instituting a national eye-care system.

**Approach:**

From 2001, the East Timor Eye Program coordinated donations and initially provided eye care through visiting teams. From 2005, the programme reoriented to undertake concerted workforce and infrastructure development. In 2008 full-time surgical services started in a purpose-built facility in the capital city. In 2014 we developed a clinical training pipeline for local medical graduates to become ophthalmic surgeons, comprising a local postgraduate diploma, with donor funding supporting master’s degree studies abroad.

**Local setting:**

In the population of 1.26 million, an estimated 35 300 Timorese are blind and an additional 123 500 have moderate to severe visual impairment, overwhelmingly due to cataract and uncorrected refractive error.

**Relevant changes:**

By April 2018, six Timorese doctors had completed the domestic postgraduate diploma, three of whom had enrolled in master’s degree programmes. Currently, one consultant ophthalmologist, seven ophthalmic registrars, two optometrists, three refractionists and four nursing staff form a tertiary resident ophthalmic workforce, supported by an international advisor ophthalmologist and secondary eye-care workers. A recorded 12 282 ophthalmic operations and 117 590 consultations have been completed since 2001.

**Lessons learnt:**

International organizations played a pivotal role in supporting the Timorese eye health system, in an initially vulnerable setting. We highlight how transition to domestic funding can be achieved through the creation of a domestic training pipeline and integration with national institutions.

## Résumé

**Problème:**

Un violent conflit a entraîné un manque de personnel et d'infrastructure sanitaires au Timor-Leste après 2001. La disparition des secteurs de la santé et de l'enseignement supérieur a aggravé les difficultés liées à la mise en place d'un système national de soins oculaires.

**Approche:**

En 2001, le Programme oculaire du Timor-Leste a commencé à coordonner les dons et à fournir des soins oculaires par l'intermédiaire d'équipes visiteuses. En 2005, le programme a été réorienté en vue d'un développement concerté du personnel et des infrastructures. En 2008, des services chirurgicaux à plein temps ont été proposés dans un établissement spécialisé de la capitale. En 2014, nous avons créé une filière de formation clinique permettant aux diplômés locaux en médecine de devenir chirurgiens ophtalmologistes. Cette filière prépare à un diplôme local d'études universitaires supérieures et permet de suivre des études de master à l'étranger grâce à un financement versé par des donateurs.

**Environnement local:**

Le Timor-Leste compte 1,26 million d'habitants, parmi lesquels environ 35 300 sont aveugles et 123 500 souffrent d'une déficience visuelle modérée à sévère due, dans la plupart des cas, à une cataracte et à des défauts de la réfraction non corrigés.

**Changements significatifs:**

En avril 2018, six médecins timorais ont obtenu un diplôme national d'études universitaires supérieures, et trois d'entre eux se sont inscrits à des programmes de master. À l'heure actuelle, un ophtalmologiste, sept médecins en cours de spécialisation en ophtalmologie, deux optométristes, trois réfractionnistes et quatre infirmiers constituent les effectifs résidents tertiaires des services ophtalmologiques. Ils sont aidés par un conseiller international en ophtalmologie et des prestataires de soins oculaires secondaires. Depuis 2001, 12 282 opérations ophtalmologiques et 117 590 consultations ont été réalisées.

**Leçons tirées:**

Des organisations internationales ont joué un rôle essentiel dans le soutien du système timorais de santé oculaire, dans une situation initialement fragile. Nous avons montré que la transition vers un financement national est possible à travers la création d'une filière de formation nationale et une intégration dans les institutions nationales.

## Resumen

**Situación:**

El violento conflicto dejó a Timor-Leste con un personal sanitario y unas infraestructuras desmanteladas después de 2001. La ausencia de sanidad y de una educación terciaria agravó los desafíos de instituir un sistema nacional de atención ocular.

**Enfoque:**

Desde 2001, el Programa Ocular de Timor-Leste coordinó las donaciones e inicialmente brindó atención oftalmológica a través de equipos visitantes. A partir de 2005, el programa se reorientó para llevar a cabo una fuerza de trabajo coordinada y para la creación de infraestructuras. En 2008, los servicios quirúrgicos a tiempo completo comenzaron a trabajar en una instalación especialmente diseñada en la capital. En 2014 desarrollamos una cartera de capacitación clínica dirigida a graduados médicos de la zona para que se convirtieran en cirujanos oculares. Se otorgó un diploma de posgrado local con fondos de donantes que respaldaron los estudios de máster en el extranjero.

**Marco regional:**

En una población de 1,26 millones, se estima que 35.300 timorenses padecen ceguera y 123.500 más padecen alguna discapacidad visual, desde moderada a severa, principalmente debido a cataratas y a errores de refracción no corregidos.

**Cambios importantes:**

En abril de 2018, seis médicos timorenses habían completado el diploma nacional de posgrado, tres de los cuales se habían matriculado en programas de estudio de máster. Actualmente, un oftalmólogo consultor, siete especialistas oftálmicos, dos optometristas, tres refraccionistas oculares y cuatro enfermeras forman el personal terciario oftálmico residente, con el apoyo de un oftalmólogo asesor internacional y trabajadores secundarios de la atención oftálmica. Se han realizado 12.282 operaciones oculares y 117.590 consultas desde 2001.

**Lecciones aprendidas:**

Las organizaciones internacionales desempeñaron un papel fundamental en el apoyo al Sistema de Salud Ocular Timorense, en un contexto inicialmente vulnerable. Cabe destacar cómo la transición hacia una financiación interna puede lograrse a través de la creación de una cartera de capacitación nacional y la integración con las instituciones nacionales.

## ملخص

**المشكلة:**

تركت النزاعات العنيفة تيمور - ليشتي مع قوى عاملة وبنية تحتية مفككتين للرعاية الصحية بعد عام 2001. وأدي غياب القطاعات الحالية للتعليم الصحي والتعليم فوق الثانوي إلى تفاقم تحديات إنشاء نظام وطني للعناية بالعيون.

**الأسلوب:**

منذ عام 2001، قام برنامج العناية بالعيون في تيمور الشرقية بتنسيق التبرعات، وقدم عناية العيون بشكل أولي من خلال الفرق الزائرة. منذ عام 2005، تم أعادة توجيه البرنامج نحو توحيد القوى العاملة وتطوير البنية التحتية. في عام 2008 بدأت الخدمات الجراحية بدوام كامل في منشأة بنيت خصيصاً لهذا الغرض في العاصمة. في عام 2014، قمنا بتطوير خط تدريب سريري لخريجي الطب المحليين ليصبحوا جراحين في طب العيون، وتألف هذا الخط التدريبي من دبلومة محلية للدراسات العليا، مع تمويل من التبرعات لدعم دراسات درجة الماجستير في الخارج.

**المواقع المحلية:**

من إجمالي عدد سكان يبلغ 1.26 مليون نسمة في تيمور الشرقية، منهم 35300 مصابين بالعمى، و123500 آخرين يعانون من ضعف في البصر معتدل إلى شديد، ويعود ذلك بدرجة كبيرة إلى الكتاراكت (إعتام عدسة العين)، وعدم تصحيح خلل الانكسار.

**التغيّرات ذات الصلة:**

بحلول شهر نيسان/أبريل 2018، أكمل ستة أطباء تيموريين دبلومة الدراسات العليا المحلية، ثلاثة منهم قاموا بالتسجيل في برامج الحصول على درجة الماجستير. وأصبح الآن هناك طبيب استشاري واحد للعيون، وسبعة من المسجلين لدراسة العيون، واثنان من أخصائيي تصحيح البصر، وثلاثة من خبراء تصحيح الانكسار، وأربعة من طاقم التمريض، يشكلون جميعاً قوة عمل مقيمة فوق الثانوية لطب العيون، يدعمها طبيب عيون استشاري دولي وعمال عناية ثانوية للعين. وقد تم الانتهاء من 12282 عملية جراحية مسجلة للعيون، فضلاً عن 117590 استشارة طبية منذ عام 2001.

**الدروس المستفادة:**

لعبت المنظمات الدولية دوراً محورياً في دعم نظام صحة العيون في تيمور، في بيئة ضعيفة في الأساس. لقد أوضحنا كيف يمكن تحقيق التحول إلى التمويل المحلي من خلال إنشاء خط تدريب محلي والتكامل مع المؤسسات الوطنية.

## 摘要

**问题:**

2001 年后，东帝汶的暴力冲突使得当地医疗人力流失，基础设施遭受重创。现有卫生部门和高等教育部门的缺失加剧了建立国家眼保健系统的挑战。

**方法:**

自 2001 年起，东帝汶眼部计划协调捐款，并首次提出由访问团队提供眼部治疗。自 2005 年起，该计划再次调整，以协调人力、发展基础设施。2008 年，首都有专门医疗机构开始提供专职外科服务。2014 年，我们开发了一条临床培训渠道，旨在培养当地医学毕业生成为眼科医师，其中包括取得当地研究生文凭，得到捐赠人资助前往海外攻读硕士学位。

**当地状况:**

在 126 万东帝汶人中，约 35300 人患有盲症，123500 人有中度至重度视力损害。未矫正的屈光不正、未经手术治疗的白内障是导致视力损害的主要原因。

**相关变化:**

截至 2018 年 4 月，六名东帝汶医生已取得国内硕士文凭，其中三人已注册入读硕士学位课程。目前，由国际顾问眼科医师和二级眼科医师支持，一名眼科专家顾问、七名执业注册眼科医师、两名视光师、三名验光师和四名护理人员组成了三级常驻眼科团队。据记载，自 2001 年起，该团队已完成 12282 次眼科手术、117590 次眼科咨询。

**经验教训:**

在初期艰苦环境中，国际组织在支持东帝汶眼保健系统方面发挥了关键作用。我们强调通过建立国内培训渠道、并与国家机构进行整合来实现国内资金的转变。****

## Резюме

**Проблема:**

Кровопролитный конфликт в Тимор-Лешти привел к разрушению системы здравоохранения и потере специалистов после 2001 года. Отсутствие действующего сектора здравоохранения и высшего образования усугубляет проблемы, связанные с созданием национальной системы офтальмологической помощи.

**Подход:**

Начиная с 2001 года действует программа по лечению глазных заболеваний для жителей Тимор-Лешти, в рамках которой осуществлялась координация пожертвований и предоставление первичной офтальмологической помощи выездными бригадами специалистов. С 2005 года программа переориентирована на совместную работу и развитие инфраструктуры. В 2008 году началось предоставление полноценных хирургических услуг в специально построенном для этого здании в столице. В 2014 году был разработан процесс клинической подготовки специалистов, чтобы выпускники местных медицинских институтов могли стать офтальмологическими хирургами. Предусмотрено также получение местного диплома о втором высшем образовании. Это становится возможным благодаря донорскому финансированию, поддерживающему обучение на магистерских программах за рубежом.

**Местные условия:**

При размере населения 1,26 млн человек около 35 300 жителей Тимора страдают слепотой, а еще 123 500 имеют нарушения зрения средней и высокой степени в основном в результате катаракты и некорректированных аномалий рефракции.

**Осуществленные перемены:**

К апрелю 2018 года шесть врачей Тимора получили местный диплом о втором высшем образовании, три из них поступили в магистратуру. В настоящее время в число местных сотрудников офтальмологических служб с высшим образованием входит один офтальмолог-консультант, семь ординаторов-офтальмологов, два оптометриста, три рефракциониста и четыре медсестры, которые работают при поддержке международного консультанта-офтальмолога и специалистов-офтальмологов среднего звена. С 2001 года было осуществлено 12 282 офтальмологические операции и 117 590 консультаций.

**Выводы:**

Международные организации сыграли ключевую роль в оказании поддержки системе офтальмологической помощи Тимора в изначально неблагоприятных условиях. Мы подчеркиваем, что за счет организации учебного процесса на местах и интеграции с национальными учреждениями может быть достигнут переход на внутреннее финансирование.****

## Introduction

Blindness and moderate-to-severe visual impairment were estimated to affect 36.0 million and 217 million people, respectively, worldwide in 2015.^1^ Of these, about 90.0% of affected people were in developing countries alone.^2^ Age-related cataract and uncorrected refractive error account for 20 million of 36 million (55.6%) cases of blindness (presenting visual acuity < 3/60 in the better eye) and 169 million of 217 million (77.9%) cases with moderate-to-severe visual impairment (presenting visual acuity > 3/60 and < 6/18 in the better eye) worldwide.^1^ Estimates suggest that nearly 80.0% of visual impairment is preventable or treatable through appropriate interventions.^2^

## Local setting

Timor-Leste is a mountainous island nation with a population of 1.26 million^3^ and is ranked 133rd globally on the 2016 Human Development Index.^4^ Approximately two-thirds of Timorese live rurally.^3^ All-cause blindness affects 35 280 people nationwide, where cataract causes 79.4% (28 000) of cases. Moderate-to-severe visual impairment affects 122 220 people and 70.0% (85 554) cases and 26.9% cases (32 914) are due to cataract and uncorrected refractive error, respectively. These numbers indicate that at least 93.0% (146 468/157 500) of all disabling visual impairment in Timor-Leste is treatable.^5^ Currently, there is one national referral hospital in the capital city, Díli, and five secondary referral hospitals with inpatient capability, in addition to 91 peripheral centres.^6^ The health-care system is provided free of charge to all citizens. Overall health-care expenditure, however, remains among the lowest reported worldwide at 1.5% of the 4 billion United States dollars (US$) gross domestic product in 2014.^3^

Violent conflict in Timor-Leste from 1999 to 2001 left a dismantled health-care workforce and infrastructure.^3^^,^^7^^–^^9^ In response to this humanitarian crisis, and at the request of World Health Organization (WHO) representatives, the East Timor Eye Program was established in July 2000. Early involvement of the Royal Australasian College of Surgeons provided organizational capacity to the programme. The sole aim of the programme from inception until 2005 was to deliver surgical interventions through visiting teams, addressing the overwhelming burden of ocular trauma and, later, the backlog of untreated cataract and refractive error.

## Approach

**Fig. 1:**
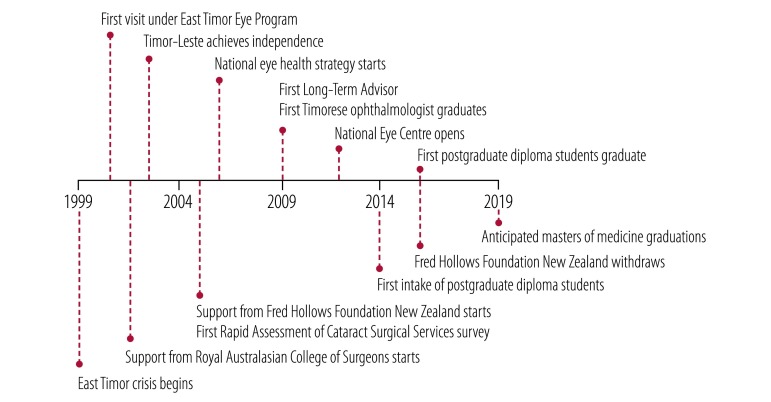
Timeline of events in the East Timor Eye Program, 1999–2020

Note: We anticipate completion of handover of the programme to the health ministry in 2020.

In 2005, the eye programme reoriented to undertake concerted workforce and infrastructure development (Fig. 1). WHO assisted by identifying international partners for the development of training pathways for medical, nursing and allied health-care staff. A rapid assessment of cataract surgical services survey was undertaken in 2005,^10^ informing the 2006 national eye-health strategy.^11^ From 2005 to 2016, the Fred Hollows Foundation New Zealand provided a crucial labour force, technical expertise and a robust funding stream.^12^ Vision2020 through Australian Aid provided support for a long-term advisor, and international training of graduates and staff. Before these arrangements the programme had been reliant on generous ad hoc funding from external donors.

From 2005 onwards, visiting specialist teams focused on vocational training of local tertiary surgical staff and collaborative development of models of care appropriate to the Timorese setting. Between visits, ophthalmology services were maintained by one Timorese doctor, who, with funding from international partners, completed an international master’s degree of medicine in ophthalmology from the University of Sydney, Australia in 2008. From then on full-time ophthalmic surgical services became available at the national referral hospital in the capital city, Díli (Fig. 1). The programme concurrently funded an experienced expatriate ophthalmologist, acting as a long-term advisor to oversee ongoing training and service development (Table 1). In July 2011 a self-contained ophthalmology centre, the National Eye Centre, was opened in the grounds of the national referral hospital. Programme partners were responsible for its construction and equipping, with the centre being wholly owned by the health ministry.

**Table 1:** Core aspects of a training pathway for ophthalmologists in Timor-Leste

Element	Relevance	Specifics	Funding
Postgraduate diploma of ophthalmology	Facilitates: foundational ophthalmic education; graduation of cataract surgeons for secondary surgical referral centres; pathway for Cuban-trained junior doctors to gain specialization	An 18-month programme with an additional 6-month induction to improve English-language ability. Senior registrars form majority of ophthalmic workforce in the capital city, Díli	Trainees funded as clinicians by health ministry
Trainee selection	Allows selection of highly engaged, academic medical graduates, helping to ensure programme success	Selection involves: written examination; interview; outstanding undergraduate performance. Approximately three graduates admitted annually	Health ministry and Timor-Leste’s national university (Universidade Nacional de Timor Lorosa’e)
Long-term advisor	Provides: tutoring for postgraduate diploma of ophthalmology; procedural support; strategic guidance for the department of ophthalmology. Need for position will cease once department’s activities are transferred to full health ministry funding	Requires: full-time position; fixed-term contract; highly-experienced clinician with experience in resource-constrained settings; necessarily foreign and has international expertise	East Timor Eye Program through grants and donors
International master’s degree of medicine in ophthalmology	Provides for: advanced ophthalmic training that cannot be delivered domestically; exposure to world-class institutes; high-caseload vocational learning; development of robust professional Timorese ophthalmic care sector; graduation of general ophthalmologists to lead secondary surgical centres	Undertaken in Fiji (Pacific Eye Institute) or Nepal (National Academy of Medical Science). Three-year course. Costs approximately US$ 120 000 per graduate	Fred Hollows Foundation New Zealand (Fiji), or East Timor Eye Program through grants and donors (Nepal)
Intensive international attachments	Provides specifically for trainees and graduates of postgraduate diploma of ophthalmology. Provides caseload required for graduation of competent Timorese cataract surgeons and subspecialization	Undertaken in Nepal. Scope of attachment limited to specific skill-set (e.g. manual small incision cataract surgery, vitreoretinal, corneal, glaucoma)	East Timor Eye Program through grants and donors
Visiting international faculty	Provides: specialist capacity-building; specialist and sub-specialist services with the aim of skills transfer; potential to reduce international case referrals	Faculty are recruited by or apply to the East Timor Eye Program. Overt purpose is to supplement the teaching curriculum	Self-funded or subsidized by the East Timor Eye Program through grants and donors

US$: United States dollars.

From 2007, the Fred Hollows Foundation New Zealand facilitated the training of dedicated primary eye-care workers, yielding a current workforce of 20 mid-level eye-care workers and five refractionists. Expanding this specialized workforce, however, has proved challenging.^12^ Since 2010, around 1000 Cuban-trained primary-care physicians have graduated to work in communities throughout the country. This initiative provides an opportunity for the development of a cost–effective workforce for the primary assessment and referral of ocular disease. In response, since 2013 the Royal Australasian College of Surgeons has delivered family medicine training, incorporating 4- to 6-week rotations at the department of ophthalmology.^8^

In 2014 the postgraduate diploma of ophthalmology was established in collaboration with Timor-Leste’s national university. Instituting a training programme was dependent on stable clinical caseload and clinical protocols appropriate to local needs. Nine registrars have undertaken attachments at international centres to provide for task-specific intensive postgraduate training (Table 1). Donors additionally provide funding for postgraduate diploma graduates to undertake a master’s degree of medicine in ophthalmology, at a cost of US$ 120 000 per trainee.

## Relevant changes

By April 2018, the resident ophthalmic workforce in Timor-Leste comprised one consultant ophthalmologist, seven ophthalmic registrars, two optometrists, three refractionists and four nursing staff, supported by the international advisor ophthalmologist. Six candidates have completed the postgraduate diploma of ophthalmology, three of whom are studying for a master’s degree of medicine in ophthalmology in Nepal or Fiji and are expected to graduate in late 2019. The remaining three graduates make up the domestic workforce as senior registrars, benefiting from both domestic and international vocational clinical teaching (Table 1).

**Fig. 2:**
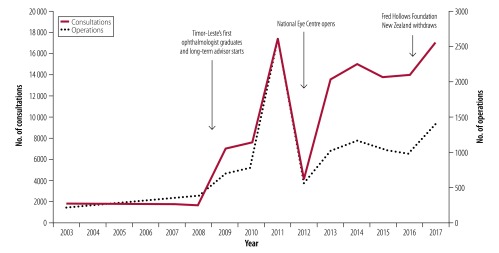
Consultations and operations per annum in Timor-Leste, 2003–2017

A total of 12 282 ophthalmic operations and 117 590 consultations have been completed since 2001 (Fig. 2). A sharp drop in consultations and operations in 2012 corresponded to the opening of the National Eye Centre, when the work of the ophthalmology department had to be diverted to achieve this. A strong relationship with the national referral hospital and the health ministry lessened the impact of the withdrawal of Fred Hollows Foundation New Zealand in late 2016, with no change in annual caseload. However, focusing on maintenance of caseload during withdrawal of international partners resulted in the decline of several systems, including electronic medical record and stock management systems, which have proved difficult to reintroduce due to a lack of endogenous expertise.

## Lessons learnt

Although the Timorese post-conflict setting is unique, we believe lessons learnt are broadly applicable to other settings. Starting with a high-volume intermittent service delivery approach facilitates the development of local, context-appropriate protocols and training of allied staff (Box 1). Initial focus on local capacity-building in preference to infrastructure provision ensures that donated goods are not underutilized. Establishing, in initial phases, that transition to local ownership is the end goal is crucial. Integrating into local health-care facilities through the guidance of a long-term advisor facilitates development of a full-time clinical service, permitting the specialist training of local medical graduates. Training a mixture of both cataract surgeons and general ophthalmologists balances the cost of advanced training against having a context-appropriate service. Elements of this model have been applied to Sumba, Indonesia,^13^ and to a new eye care programme in the Federated States of Micronesia.^14^

Box 1 **Summary of main lessons learnt**

• Continuity and stable development of eye services was ensured by the presence of a community-sector volunteer-driven eye programme to advocate for and support the development of the nascent clinical service.

• A long-term advisor with appropriate experience who acted as an instructor for physician trainees and guided the replication of effective models of care was critical for success of the training programme.

• Prerequisites for the creation of a domestic training programme were the presence of national medical graduates, robust tertiary education institutions, predictable clinical caseloads and tertiary-qualified local staff.

Improving surgical coverage of cataract and refractive error in Timor-Leste is an ongoing challenge. Over 45% of people with unoperated cataract and severe visual impairment identify access as the main barrier to care.^5^ Most Timorese still seek medical services by foot, with many walking over two hours to reach health outposts.^6^ For those with poor vision or impaired mobility this may not be possible. One-fifth of Timorese (23 of 121 surveyed) with disabling cataract did not feel the need for treatment, despite knowledge of availability and acceptability of treatment services;^5^ this could in part be due to the perception that poor vision is a normal part of ageing. Since 2005, monthly outreach trips from the capital city to regional districts have provided in situ management of refractive errors and operative management of cataract to overcome barriers to care. In 2017, outreach accounted for 442 of 1394 (31.7%) of the ophthalmology department’s surgical caseload, which suggests this is an effective mechanism that could be strengthened through funding and health promotion. Strengthening non-specialist primary eye care and establishing regional surgical centres would further address this barrier.

Presently there is one ophthalmologist in Timor-Leste, contrasting with the Vision2020 workforce recommendation of at least 25 in-country ophthalmologists.^15^ For a nation of Timor-Leste’s size, Vision2020 recommends 25 mid-level eye-care personnel and 13 refractionists. The ophthalmology department will inform the development of a 30-year plan for eye care in Timor-Leste as part of a system-wide undertaking to ensure service longevity and equality of access. The aim is to establish five cataract surgical centres staffed by future ophthalmology graduates and additional secondary non-surgical referral locations staffed by specialist mid-level eye-care workers, and supported by referrals from community-based primary medical practitioners. 

Threats to sustainability of the programme include maintenance of the training pipeline and management of conditions typically reserved for subspecialist ophthalmologists. The sustainability of the current postgraduate diploma is assured by the presence of a long-term advisor acting as an instructor. Once the international master’s degree graduates return to Timor-Leste, it is anticipated they will assume similar teaching roles. Sustainability of the international master’s degree component presents a threat to the current training pipeline given the high cost, which is currently borne by grants and philanthropic donors. A domestically delivered master’s degree is unlikely to be of long-term advantage, as the workforce recommendation for Timor-Leste^15^ corresponds to an average of one trainee per year, assuming a postgraduate career of approximately 25 years. The lack of dedicated sub-specialist ophthalmologists (e.g. specialized in vitreoretinal and orbital diseases) in Timor-Leste necessitates referring patients to neighbouring South-East Asian nations. We aim to continue to mitigate this expense by inviting visiting sub-specialists whose estimated direct costs are approximately US$ 3000 per visit. While this arrangement is cost–effective both for training and when averaged across patients requiring sub-specialist attention, reliance on this pro-bono arrangement is unlikely to be sustainable in the long term. Intensive training attachments abroad, however, provide a sustainable path for the management of sub-specialist conditions.

By prior agreement, Timorese medical and nursing personnel were always employees of the health ministry, which has proved critical for sustainable service transition. Initial provision of appropriate training to in-country staff ensured that aid and in-kind donations of equipment were used effectively. Currently, in 2018, consumables and medications are largely funded by the Timorese government. The health ministry will continue to subsume costs associated with the activities of the ophthalmology department, guided by a clear timeframe for a planned transition to full Timorese funding of the department’s activities by 2020.
